# Factors affecting recurrence in subacute granulomatous thyroiditis

**DOI:** 10.20945/2359-3997000000473

**Published:** 2022-05-12

**Authors:** Çiğdem Tura Bahadir, Merve Yilmaz, Elif Kiliçkan

**Affiliations:** 1 Amasya University Faculty of Medicine Department of Endocrinology and Metabolism Amasya Turkey Department of Endocrinology and Metabolism, Faculty of Medicine, Amasya University, Amasya, Turkey; 2 Gazi State Hospital Department of Endocrinology and Metabolism Samsun Turkey Department of Endocrinology and Metabolism, Gazi State Hospital, Samsun, Turkey; 3 Ondokuz Mayıs University Faculty of Medicine Department of Endocrinology and Metabolism Samsun Turkey Department of Endocrinology and Metabolism, Faculty of Medicine, Ondokuz Mayıs University, Samsun, Turkey

**Keywords:** Subacute thyroiditis, recurrence, prednisolone, thyrotoxicosis, risk factors

## Abstract

**Objective:**

This study aimed to evaluate the factors affecting recurrence in subacute granulomatous thyroiditis (SAT).

**Materials and methods:**

A total of 137 patients with SAT were enrolled in the study; 98 (71.5%) were women and 39 (28.5%) were men. The patients received either steroid or nonsteroidal anti-inflammatory drug (NSAID) for eight weeks. Erythrocyte sedimentation rate (ESR), C-reactive protein, serum thyroid-stimulating hormone (TSH), free triiodothyronine, free thyroxine (FT4), anti-thyroid peroxidase antibodies and thyroglobulin antibodies, neutrophil, lymphocyte, platelet, neutrophil to lymphocyte ratio, and platelet to lymphocyte ratio levels were evaluated. In addition, recurrence rates were compared between patients who received NSAID treatment and those who received steroid therapy.

**Results:**

Treatment modality and pretreatment TSH, FT4, and ESR were significantly different between patients with and without recurrence (p = 0.011, 0.001, 0.004, and 0.026, respectively). Compared with patients without recurrence, those with recurrence had higher pretreatment TSH levels, but lower FT4 and ESR levels. On logistic regression analysis, treatment modality was found to be an independent risk factor for recurrence. The risk of recurrence was higher in those taking steroids than in those taking NSAIDs (p = 0.015). The optimal TSH cutoff value for recurrence was 0.045 μIU/mL, with a sensitivity of 83.3% and specificity of 76% (AUC 0.794, 95% CI 0.639-0.949).

**Conclusions:**

The risk of SAT recurrence was higher with steroid therapy than with NSAIDs. Patients who had mild thyrotoxicosis had relatively high recurrence rate and may need a relatively longer duration of treatment.

## INTRODUCTION

Subacute granulomatous thyroiditis (SAT) is a self-limiting, inflammatory thyroid disease. It usually occurs following a viral infection and is characterized by pain in the thyroid region, thyrotoxicosis symptoms, and elevated erythrocyte sedimentation rate (ESR) and, C-reactive protein (CRP) levels (1). The characteristic appearance of SAT on thyroid ultrasound includes poorly defined margins, centripetal reduction of echogenicity, and lack of internal vascularization (2). Nonsteroidal anti-inflammatory drugs (NSAID) and steroids are used in the treatment of SAT. Patients with refractory pain were reported to have rapid relief with prednisolone treatment because of suppression of inflammation (1,3).

In daily practice, SAT may manifest with atypical presentation. Some cases have no pain in the neck, jaw, or ears; may have fever and painless swelling in the neck only; and may be euthyroid at the time of admission (4). Because SAT is not an autoimmune disorder, high levels of anti-thyroid peroxidase antibodies (anti-TPO) or thyrotropin receptor autoantibodies (TRAb) are not expected. However, in some patients the anti-TPO or TRAb concentrations may be elevated (5,6). Furthermore, some cases of SAT were reported to have a nodular instead of the typical appearance on ultrasound; this may be confused with malignancies (4,7-9). These atypical presentations may delay the diagnosis and, subsequently, the treatment of such patients with SAT.

The reason for SAT relapse remains unknown, and little is known regarding the protective and risk factors. Recent studies showed relationship of increased recurrence risk with coexistence of *Human Leukocyte Antigen-B* (HLA-B)*18:01 and HLA-B*35 and rapid tapering of prednisolone (10,11). In addition, the presence of HLA-DRB1*01 and C*04:01 were associated with genetic susceptibility to SAT (12). As protective factors, it was hypothetized that the coexistence of HLA-DRB1*15:01 and B*07:02 and the absence of HLA-A*01:01 and B*41:01 are protective against SAT recurrence and steroid dependence (13). On the other hand, no association was found between recurrence and clinical symptoms (i.e., neck pain, ear pain, and fever) or thyroid ultrasound findings at the time of onset (11,14,15).

Patients who develop SAT recurrence usually present with neck pain and thyrotoxicosis symptoms. However, recurrence may have atypical presentation and vague symptoms, such as mild thyrotoxicosis, or nonspecific symptoms (i.e., weakness or dizziness) without pain.

The aim of this study was to evaluate the factors affecting recurrence in patients with SAT.

## MATERIALS AND METHODS

Local ethics committee approval (Ref. No.: B.30.2.ODM.0.20.08/242) was obtained from the Ondokuz Mayıs University ethics committee. The study was conducted in accordance with the Declaration of Helsinki.

Adult patients who were diagnosed as SAT between January 2008 and January 2020 were included in this study. Patients with neck, jaw, or ear pain; fever; painless swelling in the neck, and/or thyrotoxicosis and the presence of hypoechoic areas with blurred margin and decreased vascularization on thyroid ultrasound, elevated ESR, and CRP were diagnosed as SAT. The following patients were excluded: 1) those who were pregnant; 2) those with malignant disease, acute or chronic inflammatory disease, autoimmune disease, severe renal, pulmonary, or liver disease, infectious disease, diabetes mellitus, and morbid obesity; and 3) those with missing data. Data were obtained from the computerized patient databases of the participating centers. The demographic characteristics, comorbidities, drugs taken, and laboratory findings were gathered from the files of the patients included. Among the routinely performed tests for the follow-up of patients with SAT, the following values were recorded: ESR, CRP, serum thyroid-stimulating hormone (TSH), free triiodothyronine (FT3), free thyroxine (FT4), anti-TPO and thyroglobulin antibodies (TgAb), neutrophil (Neu), lymphocyte (Lym), and platelet (PLT). Neutrophil to lymphocyte ratio (NLR) was calculated by dividing the total neutrophil count by the total lymphocyte count. Platelet to lymphocyte ratio (PLR) was calculated by dividing the total platelet count by the total lymphocyte count.

We divided the SAT cases into mild and severe based on the criteria defined by Benbassat and cols. (16). Mild SAT was defined when all of the following criteria were present: fever < 38 °C, absent or mild neck pain, no goiter on ultrasound or by palpation, and ESR < 60 mm/h. SAT was defined as severe in the presence of any of the following: fever > 38 °C, severe neck pain, presence of goiter on ultrasound and palpation, and ESR > 60 mm/h. Patients with mild SAT were treated with NSAID and those with moderate or severe SAT were given steroid treatment. In addition, steroid treatment was started in patients who had persistent pain and clinical symptoms after two weeks of NSAID treatment. Propranolol at a dose of 40 mg/day was initiated for patients with thyrotoxicosis symptoms. For NSAID therapy, 800-1,200 mg/day of ibuprofen in divided doses was given. For steroid therapy, 32 mg/day of methylprednisolone was given for the first 10 days and was gradually stopped over eight weeks by tapering 4 mg a week. Dose titration was adjusted according to the patient's clinical symptoms; ultrasound findings; and ESR and CRP levels. After at least eight weeks of appropriate treatment, the medication was discontinued if the clinical symptoms, thyroid function tests, and ultrasound findings improved, and the ESR and CRP levels normalized. Following treatment completion, all patients were advised of the recurrence symptoms. They were told to watch out for and return for control as needed. Otherwise, they were advised to follow-up one month later. Patients were followed-up on the 1st, 3rd, 6th, and 12th months after completion of treatment for recurrence. After discontinuation of treatment, the onset of clinical signs and symptoms, such as neck/jaw/ear pain, painless swelling in the neck, or fever, and/or thyrotoxicosis plus SAT findings on ultrasound and elevated ESR and CRP was regarded as recurrence.

Patients with and without recurrence and patients taking steroids and NSAIDs were compared in terms of demographic characteristics and laboratory findings (i.e., ESR, CRP, TSH, FT3, FT4, anti-TPO, TgAb, Neu, Lym, PLT, NLR, and PLR). The thyroid hormone levels of patients were analyzed by immunoassay (Roche Cobas-e 602, Basel, Switzerland). Hemograms were determined with an autoanalyzer (Sysmex-XN hematology system, Hamburg, Germany). The reference ranges were as follows: TSH 0.27-4.2 μIU/mL, FT4 0.93-1.7 ng/dL, FT3 2.0-4.4 pg/mL, anti-TPO < 34 IU/mL, TgAb < 115 IU/mL, ESR 0-20 mm/h, CRP 0-5 mg/L, Neu 1,650-4,970 cell/μL, Lym 1,170-3,170 cell/μL, and PLT 170,000-360,000 cell/μL.

### Statistical analysis

Data were analyzed using the Statistical Package for Social Sciences software (SPSS 18). The distribution of continuous parameters was evaluated by Kolmogorov-Smirnov and Shapiro-Wilks tests. For normally distributed continuous parameters, groups were compared using independent samples t-test, whereas non-normally distributed continuous parameters were compared by Mann-Whitney U Test. Nominal parameters were analyzed by chi-square test and Fisher's exact test. The difference between pre- and posttreatment values was evaluated by Wilcoxon test. For each risk factor, the regression coefficient was determined with 95% confidence interval and level of significance. The factors that were found to affect recurrence in the univariate analysis were included in the binary logistic regression analysis to determine the independent factors. For diagnostic purposes, the cutoff value was determined using Receiver Operating Characteristic (ROC) curve. The value corresponding to the maximum summation of sensitivity and specificity was taken as the cutoff value. Statistical significance was accepted at p < 0.05.

## RESULTS

Of the 956 patient records, a total of 137 fulfilled the inclusion criteria. The remaining 819 cases were excluded for issues of noncompliance (i.e., shorter treatment and missed follow-ups) with the treatment protocol, missing data, and the other factors described in the methods. Power analysis of this study yielded a power of 89.6%. The median age of the population was 43 years (range, 27-68 years); 98 (71.5%) were women and 39 (28.5%) were men. The clinical characteristics of the patients are summarized in Table 1.

**Tabla 1:** Demographic data and laboratory results of the patients

Parameters	Patients
Age (years)	43 (27-68)
Sex	98 (71.5%) female 39 (28.5%) male
TSH (μIU/mL)	0.01 (0.01-3.78)
FT3 (pg/mL)	5.49 (1.38-17.26)
FT4 (ng/dL)	2.39 (0.94-7.18)
Neu (cell/μL)	5,860 (2,600-14,830)
Lym (cell/μL)	2000 (600-4,610)
PLT (cell/μL)	363,503 ± 91,040
ESR (mm/h)	69.3 ± 23.4
CRP (mg/L)	46.7 (6.4-273)
NLR	2.8 (0.9-11.3)
PLR	173.3 (38-818.3)
Anti-TPO	8 (5.8%) positive 129 (94.2%) negative
TgAb	30 (21.9%) positive 107 (78.1%) negative

TSH: thyroid-stimulating hormone; FT4: free thyroxine; FT3: free triiodothyronine; Neu: neutrophil; Lym: lymphocyte; PLT: platelet; anti-TPO: anti-thyroid peroxidase antibodies; TgAb: thyroglobulin antibodies; CRP: C-reactive protein; ESR: erythrocyte sedimentation rate; NLR: neutrophil to lymphocyte ratio; PLR: platelet to lymphocyte ratio.

To convert from ng/dL to pmol/L, multiply by 12.87 for FT4.

To convert from pg/mL to pmol/L, multiply by 1.53 for FT3.

Data are expressed as median (interquartile range), number (percentage), or mean (±SD).

Sixty-five (47.4%) patients were treated with NSAIDs, and 72 (52.6%) patients were treated with steroids. Of 137 patients, 12 (8.8%) had recurrence and the remaining 125 (91.2%) patients had no recurrence. Among patients who received steroid and NSAID treatments, 11 (15.3%) and 1 (1.5%) patient, respectively, had recurrence (Table 2). Of 12 patients who developed recurrence, 11 (91.7%) received steroids and 1 (8.3%) received NSAID.

**Tabla 2:** Comparison of patients taking steroids and NSAIDs

		Steroid treatment (n = 72)	NSAID treatment (n = 65)	p
Age (years)		43 (28-67)	41 (27-68)	0.576
Sex				
	Female	55 (76.4%)	43 (66.2%)	0.256
	Male	17 (23.6%)	22 (33.8%)	
TSH (μIU/mL)		0.01 (0.01-2.38)	0.01 (0.01-3.78)	0.203
FT3 (pg/mL)		5.26 (1.38-15.4)	5.59 (2.09-17.26)	0.943
FT4 (ng/dL)		2.29 (0.94-7.18)	2.45 (1.04-6.26)	0.904
Neu (cell/μL)		6,225 ± 2,015	5,718 ± 1,836	0.127
Lym (cell/μL)		1,930 (870-4,610)	2010 (600-3,660)	0.465
PLT (cell/μL)		354,041 ± 92,608)	373,984 ± 88,803	0.202
ESR (mm/h)		72.8 ± 24.9	65.5 ± 21.1	0.068
CRP (mg/L)		46 (6.4-273)	46.7 (8-201)	0.850
NLR		3 (1.21-6.26)	2.73 (0.91-11.33)	0.038
PLR		178.6 (38-363.6)	165.8 (82.5-818.3)	0.829
Anti-TPO				
	Positive	1 (1.4%)	7 (10.8%)	0.027
	Negative	71 (98.6%)	58 (89.2%)	
TgAb				
	Positive	11 (15.3%)	19 (29.2%)	0.078
	Negative	61 (84.7%)	46 (70.8%)	
Recurrence rate		11 (15.3%)	1 (1.5%)	0.011

NSAID: nonsteroidal anti-inflammatory drug; TSH: thyroid-stimulating hormone; FT3: free triiodothyronine; FT4: free thyroxine; Neu: neutrophil; Lym: lymphocyte; PLT: platelet; ESR: erythrocyte sedimentation rate; CRP: C-reactive protein; NLR: neutrophil to lymphocyte ratio; PLR: platelet to lymphocyte ratio; anti-TPO: anti-thyroid peroxidase antibodies; TgAb: thyroglobulin antibodies.

To convert from ng/dL to pmol/L, multiply by 12.87 for FT4.

To convert from pg/mL to pmol/L, multiply by 1.53 for FT3.

Data are expressed as median (interquartile range), number (percentage), or mean (±SD); p < 0.05.

As shown in Table 3, patients with and without recurrence had significantly different treatment modality (p = 0.011), pretreatment TSH (p = 0.001), FT4 (p = 0.004), and ESR (p = 0.026). Compared with patients without recurrence, those with recurrence had higher TSH levels, but lower FT4 and ESR levels. On the other hand, age, sex, FT3, anti-TPO, TgAb, CRP, Neu, Lym, PLT, NLR, and PLR were not different between patients with and without recurrence.

**Tabla 3:** Comparison of patients with and without recurrence before treatment

Parameters		Recurrence (n = 12)	Nonrecurrence (n = 125)	p
Age (year)		41 (32-54)	43 (27-68)	0.575
Sex				
	Female	11 (91.7%)	87 (69.6%)	0.178
	Male	1 (8.3%)	38 (30.4%)	
TSH (μIU/mL)		0.3 (0.01-2.38)	0.01 (0.01-3.78)	0.001
FT3 (pg/mL)		4.48 (2.91-9.3)	5.5 (1.38-17.26)	0.21
FT4 (ng/dL)		1.47 (0.94-4.35)	2.44 (1.04-7.18)	0.004
Neu (cell/μL)		6,360 (3,940-8,228)	5,830 (2,600-14,830)	0.422
Lym (cell/μL)		1,910 (1200-2960)	2,000 (600-4,610)	0.746
PLT (cell/μL)		339,833 ± 86,512	365,776 ± 9,1471	0.348
ESR (mm/h)		55 ± 18.2	70.7 ± 23.4	0.026
CRP (mg/L)		48.5 (6.4-88)	46.7 (7-273)	0.81
NLR		3.03 (2.03-5.58)	2.77 (0.91-11.33)	0.319
PLR		178 (91.9-226.8)	169.7 (38-818.3)	0.897
Anti-TPO				
	Positive	0 (0.0%)	8 (6.4%)	1.0
	Negative	12 (100.0%)	117 (93.6%)	
TgAb				
	Positive	2 (16.7%)	28 (22.4%)	1.0
	Negative	10 (83.3%)	97 (77.6%)	
Treatment				
	Steroid	11 (91.7%)	61 (48.8%)	0.011
	NSAID	1 (8.3%)	64 (51.2%)	

TSH: thyroid-stimulating hormone; FT4: free thyroxine; FT3: free triiodothyronine; Neu: neutrophil; Lym: lymphocyte; PLT: platelet; CRP: C-reactive protein; ESR: erythrocyte sedimentation rate; NLR: neutrophil to lymphocyte ratio; PLR: platelet to lymphocyte ratio; NSAID: nonsteroidal anti-inflammatory drug.

To convert from ng/dL to pmol/L, multiply by 12.87 for FT4.

To convert from pg/mL to pmol/L, multiply by 1.53 for FT3.

Data are expressed as median (interquartile range), number (percentage), or mean (±SD); p < 0.05.

**Figure 1:**
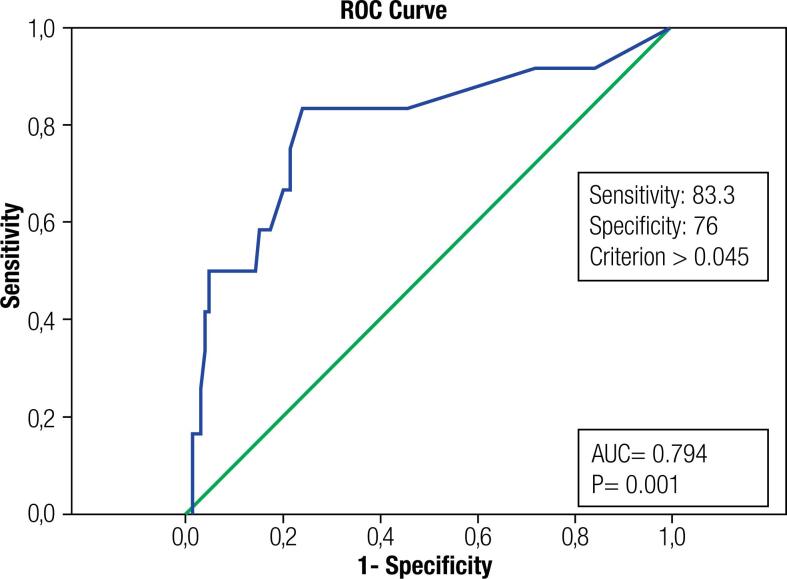
Receiver operating characteristic (ROC) curve analysis for thyroid-stimulating hormone (TSH). AUC: area under curve.

On logistic regression analysis, treatment modality was found to be an independent risk factor for recurrence. The risk of recurrence was higher in those taking steroids than in those taking NSAIDs (OR 23.003, 95% CI 1.828–289.490, p = 0.015) (Table 4). The optimal TSH cutoff value for recurrence was 0.045 μIU/mL, with sensitivity of 83.3% and specificity of 76% (AUC 0.794, 95% CI 0.639-0.949) (Figure 1). Of all recurrence cases (n = 12), 10 (83.3%) had TSH > 0.045 μIU/mL.

**Tabla 4:** Logistic regression analysis of the risk factors for recurrence

Variables	Beta	Sig	Odds Ratio	95% CI	
				Lower	Upper
TSH	0.995	0.057	2.705	0.969	7.548
FT4	-0.410	0.368	0.664	0.272	1.619
ESR	-0.019	0.262	0.981	0.949	1.014
Treatment modality*	3.136	0.015	23.003	1.828	289.490
Constant	-2.946	0.113	0.053		

* NSAID treatment was designated as “0”, steroid treatment was designated as “1”.

TSH: thyroid-stimulating hormone; FT4: free thyroxine; ESR: erythrocyte sedimentation rate.

On post-hoc analysis, we reinvestigated and compared the factors between the recurrence and nonrecurrence subgroups of the population of patients who received steroid treatment. Compared with patients without recurrence, those with recurrence had significantly higher TSH and significantly lower FT3, FT4, and ESR (Table 5).

**Tabla 5:** Comparison of patients with and without recurrence wih receipt of steroid therapy

		Patients with recurrence (n = 11)	Patients without recurrence (n = 61)	p
Age (years)		42 (32-54)	44 (28-67)	0.724
Sex				0.440
	Female	10 (90.9%)	45 (73.8%)	
	Male	1 (9.1%)	16 (26.2%)	
TSH (μIU/mL)		0.12 (0.01-2.38)	0.012 (0.01-1.25)	0.003
FT3 (pg/mL)		4.47 (2.91-7.88)	5.49 (1.38-15.4)	0.049
FT4 (ng/dL)		1.55 (0.94-4.35)	2.39 (1.12-7.18)	0.008
Neu (cell/μL)		6,700 (3,940-8,228)	5900 (3300-14830)	0.673
Lym (cell/μL)		1,930 (870-4,610)	1930 (1200-2960)	0.975
PLT (cell/μL)		341,000 (190,000-465,000)	360,000 (168,000-589,000)	0.748
ESR (mm/h)		56 (34-94)	78 (23-130)	0.011
CRP (mg/L)		35 (6.4-88)	47 (7-273)	0.707
NLR		2.99 (2.03-5.58)	3.02 (1.21-6.26)	0.725
PLR		176.7 (91.9-226.8)	180 (38-363,6)	0.725
Anti-TPO				1
	Positive	0	1 (1.6%)	
	Negative	11 (100.0%)	60 (98.4%)	
TgAb				0.672
	Positive	2 (18.2%)	9 (14.8%)	
	Negative	9 (81.8%)	52 (85.2%)	

TSH: thyroid-stimulating hormone; FT3: free triiodothyronine; FT4: free thyroxine; Neu: neutrophil; Lym: lymphocyte; PLT: platelet; ESR: erythrocyte sedimentation rate; CRP: C-reactive protein; NLR: neutrophil to lymphocyte ratio; PLR: platelet to lymphocyte ratio; anti-TPO: anti-thyroid peroxidase antibodies; TgAb: thyroglobulin antibodies.

To convert from ng/dL to pmol/L, multiply by 12.87 for FT4.

To convert from pg/mL to pmol/L, multiply by 1.53 for FT3.

Data are expressed as median (interquartile range) or number (percentage); p < 0.05.

## DISCUSSION

The main finding of our study was the significantly higher recurrence rate of SAT in patients who received steroid treatment and/or had mild thyrotoxicosis.

SAT is a self-limiting thyroid disease and is considered to be a T-cell-mediated hypersensitivity reaction against follicular epithelial cells, which carry viral antigens (17-19). As a result, inflammatory acute phase reactants, such as NLR, PLR, ESR, and CRP, are elevated in SAT (1,16,20-25). For treatment, prednisolone had been used for its anti-inflammatory effects (1).

The reported recurrence rate of SAT was 1.6% to 20% despite proper treatment (1,17). In our study, the recurrence rate was 8.8% (12 patients) and was in line with that reported in literature (1,5,11,15-17,26-30). The wide variation in recurrence rates may be because of differences in study population (Caucasian vs. Asian) and size, and duration of treatment (6 weeks vs 8 weeks). In our study, despite eight weeks of appropriate treatment protocol, the recurrence rate was significantly higher in patients treated with steroids (15.3%) than in those treated with NSAID (1.5%); this result was in compliance with the literature (Table 6) (1,5,23). Kubota and cols. found that eight weeks of steroid treatment was sufficient for 80% of patients, whereas treatment longer than eight weeks was probably needed in the remaining 20% (26). Some studies reported a nonsignificant trend of higher recurrence rate with steroids than with NSAIDs (Table 7) (5,15,29). On the other hand, in our analysis, steroid treatment was found to be an independent risk factor for recurrence. This result did not mean that steroids should be avoided for treatment but underscored the importance of paying attention to some points while using steroids. First, in this study, steroid was given to patients who had severe clinical manifestations and might have required a longer treatment duration. Second, steroids might have suppressed the inflammatory response without affecting the disease process, thereby, resulting in the persistence of the subclinical inflammatory process and rebound upon cessation of steroid treatment. Detecting patients who require longer treatment may help prevent recurrences. Patients in whom steroid is discontinued after a sufficient period of treatment and normalization of clinical, laboratory, and ultrasound findings must be followed-up closely.

**Tabla 6:** Clinical characteristics and laboratory results of the patients with recurrence at the time of diagnosis

Patient	Age (years)	Sex	Symptoms	ESR (mm/h)	CRP (mg/L)	TSH (μIU/mL)	FT4 (ng/dL)	FT3 (pg/mL)	Anti-TPO	TgAb	Initial treatment	Treatment duration (weeks)	Recurrence time (weeks)	Recurrence treatment	Treatment duration (weeks)
1	36	F	Neck, ear, jaw pain	37	40	2.38	1.40	2.91	N	P	steroid	12	4	steroid + NSAID	6
2	54	F	Painless swelling in the neck	48	8	1.00	1.20	3.60	N	N	steroid	11	4	steroid + NSAID	12
3	40	F	Neck pain	38	52	0.49	1.15	9.30	N	N	NSAID	12	4	NSAID	32
4	39	M	Painful swelling in the neck	56	65	1.31	1.23	4.47	N	N	steroid	9	8	NSAID	3
5	32	F	Neck pain	77	84	0.01	2.66	7.88	N	P	steroid	8	4	observation	4
6	45	F	Neck, ear, jaw pain	34	45	2.20	0.94	3.39	N	N	steroid	8	4	NSAID	3
7	49	F	Neck pain	45	6	1.25	1.07	3.30	N	N	steroid	24	4	steroid	7
8	47	F	Neck and ear pain	60	11	0.08	1.55	4.40	N	N	steroid	8	4	steroid	4
9	35	F	Neck and ear pain	94	67	0.05	2.08	4.50	N	N	steroid	8	4	NSAID	2
10	42	F	Neck pain	61	74	0.01	4.35	6.35	N	N	steroid	12	4	steroid	4
11	43	F	Neck and ear pain	69	88	0.05	2.20	6.10	N	N	steroid	8	4	steroid	4
12	40	F	Fever, painless swelling in the neck	41	31	0.12	1.83	5.80	N	N	steroid	8	4	NSAID	3

ESR: erythrocyte sedimentation rate; CRP: C-reactive protein; TSH: thyroid-stimulating hormone; FT4: free thyroxine; FT3: free triiodothyronine; Anti-TPO: anti-thyroid peroxidase antibodies; TgAb: thyroglobulin antibodies; F: female; M: male; N: negative; P: positive; NSAID: Nonsteroidal anti-inflammatory drug.

To convert from ng/dL to pmol/L, multiply by 12.87 for FT4.

To convert from pg/mL to pmol/L, multiply by 1.53 for FT3.

**Tabla 7:** Previous reports on subacute thyroiditis in patients on steroid therapy

	Total number of patients	Number of patients taking steroids	Duration of steroid treatment (weeks)	Steroid dosage	Overall recurrence rate	Recurrence rate (steroid group)	Recurrence rate (NSAID group)	p
Kubota et al. (26), 2013	219	219	6-12	15 mg PSL				
Sencar et al. (5), 2019	217	91	6	48 mg MP	19.8%	23%	10.5%	0.306
Our study, 2020	137	72	8	32 mg MP	8.8%	15.3%	1.5%	0.011
Erdem et al. (15), 2007	169	50	6-8	35-60 mg PSL	12.4%	16%	10.9%	0.317
Mizukoshi et al. (1), 2001	36	36	5-6	25-30 mg PSL		22%		
Fatourechi et al. (27), 2003	94	34	5	30-40 mg PSL		10%		
Arao et al. (28), 2015	26	26		15-30 mg PSL	15.3%			
Sato et al. (29), 2017	42	25		15 mg PSL	9.5%	12%	5.8 %	0.635
Bennedbaek and Hegedüs (30), 1997	23	23	6-12	37.5 mg PSL	35%			
Stasiak et al. (11), 2019	49				14%			
Benbassat et al. (16), 2007	56	18			0%			
Nishihara et al. (17), 2008	852				1.6%			

NSAID: nonsteroidal anti-inflammatory drug; PSL: prednisolone; MP: methylprednisolone.

In our study, patients with recurrence had higher TSH but lower FT4 and ESR levels, compared with the levels in patients without recurrence. Our post-hoc analysis of patients who received steroid treatment revealed similar results where TSH was higher and FT4, ESR, and FT3 were lower in patients with recurrence. Stasiak and Lewiński postulated that increased thyrotoxicosis (low TSH, and high FT4 and FT3) is a result of the damage to the thyroid follicles and is predictive of a SAT course without recurrence and that more severe thyroid tissue damage may play a protective role against recurrence (31). Sencar and cols. found lower FT4 levels but no significant difference in TSH in patients with recurrence, compared with the levels in patients without recurrence. Neither of these studies found a significant difference in ESR (5,11). This situation was quite unexpected because the cases that had worse laboratory findings, had low recurrence rate, whereas the cases with mild laboratory findings had high rate of recurrence. We postulated some reasons for these results. First, some patients may have early onset of clinical manifestations but a slow and prolonged disease course. When treatment is stopped, the inflammatory parameters may increase again and may be incorrectly attributed to recurrence. Second, compared with milder cases, those with worse laboratory findings might have developed a more prominent immune response and suppressed the disease better. In this study, the median TSH level of the patients who had recurrence was 0.3 μIU/mL (range, 0.01-2.38 μIU/mL). Moreover, the optimal TSH cutoff value for prediction of recurrence was 0.045 μIU/mL, and 83.3% of our patients who developed recurrence had TSH values > 0.045 μIU/mL. Therefore, in order to prevent recurrence, longer treatment seems to be appropriate for patients receiving steroids if they have mild thyrotoxicosis at the time of diagnosis, especially those with TSH > 0.045 μIU/mL.

Anti-TPO positivity was suggested to be protective against recurrence, based on findings of relatively high anti-TPO levels in patients without recurrence (11). Stasiak and cols. regarded anti-TPO positivity as a sign of autoimmune thyroid disorder (AITD) and speculated that the HLA antigens present in AITD may be protective against SAT recurrence, contrary to those that increase SAT recurrence (11,32,33). However, in this study, we did not find any association between recurrence and anti-TPO or TgAb positivity despite the significantly higher anti-TPO percentage in the patient group that received NSAID treatment than the group that received steroid.

The reported rate of TgAb positivity in the general population was 11.5% (34), which was higher than that (21.9%) in our study population. Excessive destruction of the follicles can result in release of thyroglobulin into the systemic circulation; this antigenic exposure can lead to increased production of antibodies against them (35).

NLR was another parameter that we evaluated for its effect on SAT recurrence. The significantly high NLR in patients who received steroid treatment might be attributed to the high level of inflammation in this group of patients. However, we did not find any significant difference in NLR between patients with and without recurrence.

There were some limitations in this study. First, this was a retrospective study and we did not have control on the parameters that may have affected the results. The patients received treatment not based on randomization but on their clinical condition; this might have caused selection bias, which is unavoidable in retrospective studies. For example, ultrasound examination was performed by several endocrinologists. Second, because histopathologic examination was not performed, it was uncertain whether the anti-TPO positivity resulted from an underlying chronic thyroiditis or was an atypical presentation of SAT.

In conclusion, SAT recurrence was more common in patients who received steroids and had mild thyrotoxicosis at the time of diagnosis. Future studies may investigate the efficacy of a longer treatment duration in patients with mild thyrotoxicosis.
